# Supplementary material for: Quantum criticality at cryogenic melting of polar bubble lattices
Source: Nat Commun. 2023 Nov 30;14:7874. doi: 10.1038/s41467-023-43598-0 (PMC10689468; doi:10.1038/s41467-023-43598-0)
Supplement: Supplementary file 1 — Supplementary Information [file 41467_2023_43598_MOESM1_ESM.pdf]

Supplementary Information for "Quantum criticality at cryogenic melting of polar bubble lattices"

W. Luo[1], A. Akbarzadeh[1,2], Y. Nahas[1], S. Prokhorenko[1*] and L. Bellaiche[1*]

[1]Physics Department and Institute for Nanoscience and Engineering, University of Arkansas, Fayetteville, Arkansas 72701, USA
[2]Science, Engineering, and Geosciences, Lonestar College, 9191 Barker Cypress Road, Cypress, Texas 77433, USA

Corresponding author: prokhorenko.s@gmail.com, laurent@uark.edu

The aim of this supplementary information is to provide more details about (i) the effects of screening factors on dipolar patterns; (ii) The characterization of the bimerons-bubbles; (iii) the dynamical patterns for the bubble liquids and dipolar liquid phases under an electric field of E=90×10^7V/m and E=114×10^7V/m for P=32, respectively; (iv) the topological patterns for all considered electric fields and Trotter numbers; (v) the structure factors of topological patterns for all considered electric fields and Trotter numbers. (vi) the zeroth Betti number for topological patterns associated with our considered electric fields and Trotter numbers. (vii) Schematic diagram of quantum critical fluctuations and (viii) The dielectric response from CMC and PI-QMC simulations.

Supplementary Note 1. The effects of screening factors on dipolar patterns

Topological patterns can be related with the screening factor. There are two extreme cases. The first is the ideal open-circuit (OC) condition, for which unscreened polarization-induced surface charges can generate a large depolarizing electric field along the growth direction. The second one is the ideal short-circuit (SC) condition, which is associated with a vanishing internal field resulting from the full screening of surface charges The OC and SC conditions are characterized by $\beta_{oc}(\beta_{oc}$=0) and $\beta_{sc}(\beta_{sc} = 1)$ in our methodology. Previous works[1] indicate that below a certain value

of β close to 85% for PZT film under compressive (-2.65%) strain, the results become independent of β (since the initial depolarizing field is large enough to not affect anymore properties, as a result of the fact that dipoles have adopted striking patterns to "counter-attack" it). This is confirmed by the calculations shown below in Fig. 3, in that the results of β=80% (which is the β used in the present work) are similar to those of β=70% and 60%, both for CMC (P=1), that gives labyrinths pattern, and PI-QMC (P=32), that also yields labyrinths but with smaller dipoles. On the other hand, a larger β of 90% makes the labyrinths pattern disappear in favor of monodomains for CMC (and dipolar liquid for PI-QMC), as consistent with short-circuit-like conditions. In that regime of larger β, the magnitude of the dipoles significantly depends on β since the residual depolarizing field is rather small within the monodomain but strongly dependent on this screening parameter.

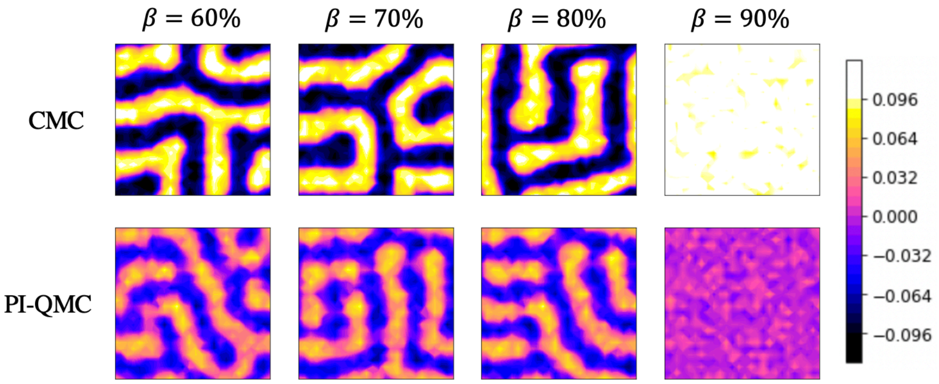

FIG. S1 (a) The topological patterns for different screening factor from CMC (P=1) and PI-QMC (P=32). The color bar indicates the z-component of the local modes u_z (in atomic units).

Supplementary Note 2. The characterization of the bimerons-bubbles

Here, we calculated the dipolar structure for the z=4 plane of the supercell of the PZT film (under electric field E=24×10^{7}V/m with P=1). The results are shown in Fig. S2a. The magnitude of the arrows represents the in-plane components. The color relates to the magnitude of the out-of-plane components. To characterize the topological properties of bimerons and bubbles, we numerically computed the skyrmion charge density (ρ_{sk}) based on the formula $q = \frac{1}{4\pi}\boldsymbol{u} \cdot (\partial_x \boldsymbol{u} \times \partial_y \boldsymbol{u})$ adapted to vector fields on the lattice with the Berg and Luscher approach [2] for each unit cell of the PZT film (under electric field E=24×10^{7}V/m with P=1). The results are shown in Fig. S2b. One can clearly see that the skyrmion charge density is broken into two parts (blue rectangles), each corresponding to a ½ charged meron.

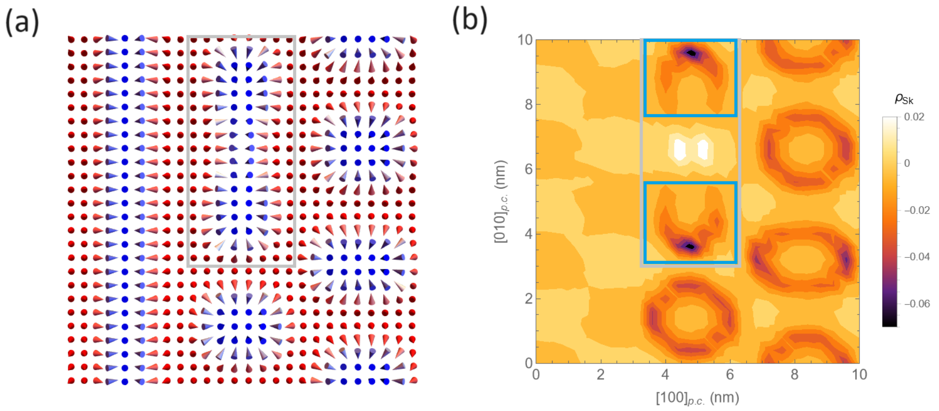

FIG. S2 The dipolar structure (a) and skyrmion charge density (ρ_{sk}) (b) for the z=4 plane of the supercell of the PZT film (under electric field E=24×10^{7}V/m with P=1). The bimeron pattern is indicated by a gray rectangle in (a) and (b). One can clearly see that the skyrmion charge density is broken into two parts (blue rectangles), each corresponding to a ½ charged meron.

Supplementary Note 3. The dynamic patterns for the bubble liquids and dipolar liquid phase (for P=32) under electric field E=90×10^{7}V/m and E=114×10^{7}V/m.

Fig. S3a shows the evolution of the bubble liquids (E=90×10^7V/m) as a function of MC sweeps, at 20 K for P=32. The total number of MC sweeps is 200,000, and we concentrate on the last 50,000 MC sweeps to make such figure. More precisely, Fig. S3a includes 20 different images among these last 50,000 MC sweeps, with the interval between each image being 2,500 MC sweeps. Similarly, Fig. S3b shows the corresponding evolution of the dipolar liquid state (for E=114×10^7V/m) as a function of MC sweeps and at 20 K for P=32. One can see easily that both the bubble liquids and dipolar liquids change in morphology between different MC sweeps, indicating their dynamical character. Note that such dynamical character of the bubble liquids and dipolar liquid phase are solely caused by the QFs since these phases do not exist in the CMC case.

(a) (b) (c)

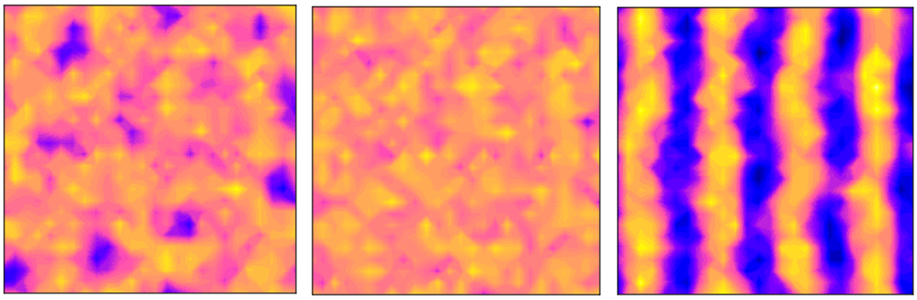

FIG. S3: (a) The evolution of the bubble liquids (for E=90×10^7V/m) as a function of MC steps (at 20 K for P=32). (b) The evolution of the dipolar liquid phase (E=114×10^7V/m) as a function of MC steps (at 20 K for P=32). (c) The evolution of the dynamic stripes (E=26×10^7V/m) as a function of MC steps (at 20 K for P=32).

Supplementary Note 4. Topological patterns for different electric fields and Trotter numbers

Fig. S4 display the topological patterns for all electric fields and Trotter numbers. For the CMC (P=1) case, and for electric fields ranging from 0×10^7V/m to 14×10^7V/m, the CMC gives connected and disconnected labyrinths. While for PI-QMC (P=32), the

connected and disconnected labyrinths have a wider range electric field values (from 0×10^7V/m to 20×10^7V/m). Further increasing electric field, the CMC yields that the labyrinths evolve into mixed bimerons-skymions, bubbles and monodomain. However, the PI-QMC (P=32) predicts very different results due to QFs. Specifically, before evolving into a mixed bimerons-skymions, the labyrinths first transform into nanostripes. Then, the nanostripes evolve into mixed bimerons-skymions, bubbles phase (42×10^7V/m to 84×10^7V/m), bubble liquids (84×10^7V/m to 94×10^7V/m), dipolar liquid phase (94×10^7V/m to 142×10^7V/m) and then monodomain, with increasing electric field. Note that, for the same electric field value, the PI-QMC computations always decreases the magnitude of the dipolar moments, as compared with CMC calculations.

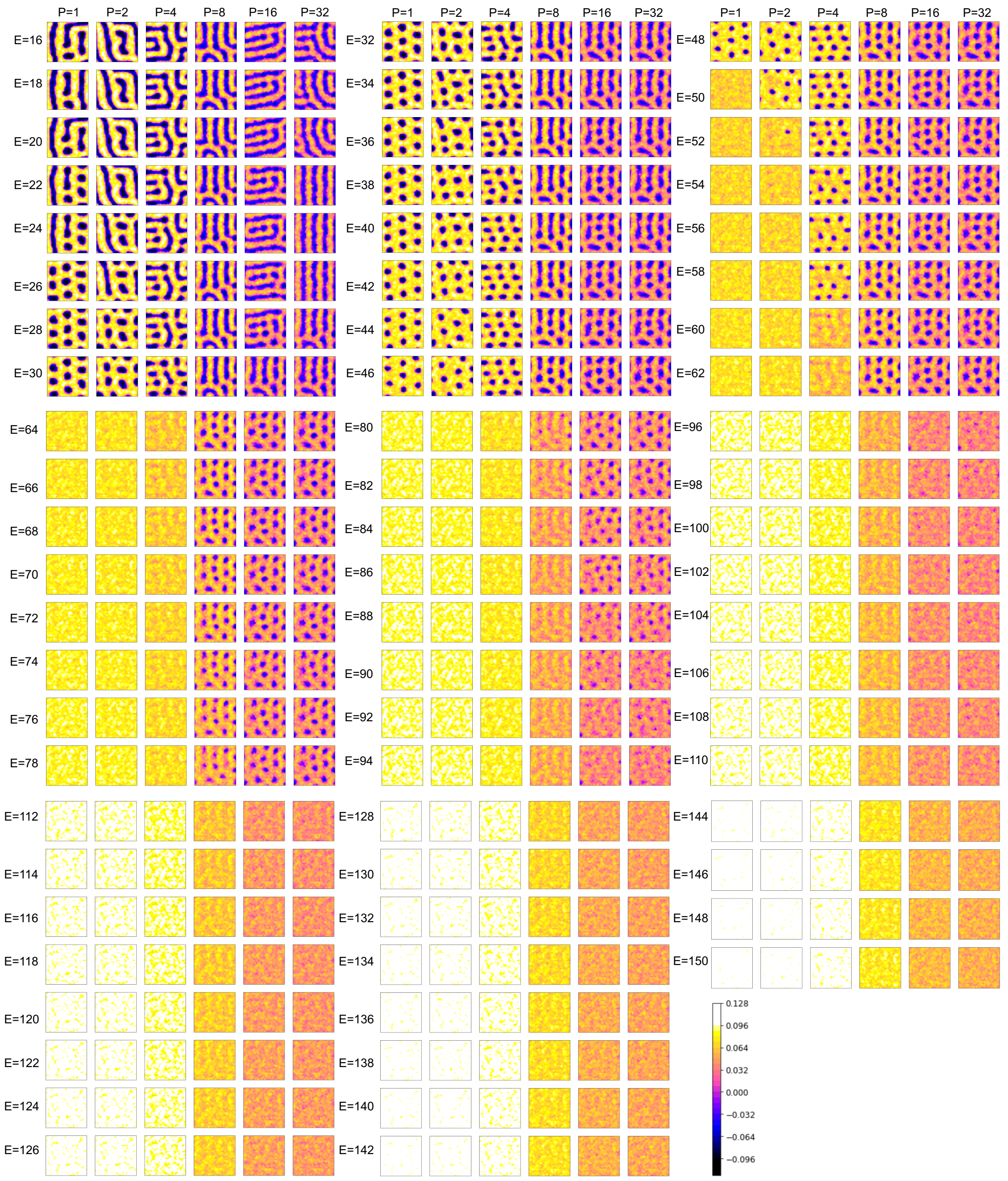

FIG. S4: topological patterns for all considered electric fields (range from 0×10^7V/m to 150×10^7V/m, with steps of 2×10^7V/m) and Trotter number P (1, 2, 4, 8, 16, 32), as obtained in a 26×26×5 supercell. The color bar indicates the z-component of the local modes u_z (in atomic units).

Supplementary Note 5. Structure factors for topological patterns under different electric fields and Trotter number.

To further analyze the inherent symmetries and orderings of different topological patterns, we calculated their structure factor $S(a\vec{k})$, with the results being shown in Fig. S5. Here, a is the lattice parameter and $\vec{k}$ (including k_x and k_y which correspond the horizontal and vertical axes in Fig. S5) is the wave vector in the reciprocal space. Practically, we get the $S(a\vec{k})$ by calculating thermodynamic average (over the last 20 topological patterns) of the squared norm (*i.e.*, we compute the intensity of structure factor $S(a\vec{k})$) of the three-dimensional discrete Fourier transform of the z component of local modes u_z (in atomic units).

For the CMC (P=1) case, for different electric fields (from 0×10^7V/m to 14×10^7V/m), as compared with connected labyrinths, one can see that there is an additional peak for the disconnected labyrinths in the center of the reciprocal space (for example, compare the black arrow in E=0×10^7V/m and E=14×10^7V/m for P=1 in Fig. S5). With increasing electric field, the phase is mixed bimerons-bubbles whose structure factors (red color) start to form more fully as compared to disconnected labyrinths (for example, compare the E=10×10^7V/m and E=16×10^7V/m for P=1 in Fig. S5), indicating that the mixed bimerons-bubbles are more isotropic than the disconnected labyrinths. Interestingly, the patterns have a trend to become vertical nanostripes (though these stripes do not fully form) which is indicated by the horizonal dark blue region (see the black solid square in E=14×10^7V/m and P=1 in Fig. S5). When further increasing the electric field, the structure factor becomes more isotropic (see E=40×10^7V/m and P=1 in Fig. S5 for example), indicating the bubble phase. For the case of (E=50×10^7V/m, P=1), there is only one central peak for the structure factor, as consistent with the formation of a monodomain (dipolar moments are along the $+z$ direction [yellow color in Fig. S4]) in the real space.

For the PI-QMC (P=32) case, as compared with the CMC case, at low electric fields (from E=0×10^7V/m to E=20×10^7V/m), one can see that the size of the red peak (which corresponds to the dipolar moments being along the $-z$ direction (blue color) in real space, while the electric field direction is along $+z$) is smaller than that of CMC, indicating that the domain size (made by $-z$ dipolar moments, *i.e.* blue color for topological patterns in real space) from PI-QMC is smaller than that of CMC. For an electric field E=24×10^7V/m (P=32), there are two peaks located at left and right sides (see black arrows in Fig. S5), indicating the existence of vertical nanostripes. For the mixed bimerons-bubbles (from E=28×10^7V/m to E=42×10^7V/m), and as similar to the CMC case, their structure factors start to form more fully as compared with disconnected labyrinths (for example, compare the E=16×10^7V/m and E=32×10^7V/m for P=32 in Fig. S5), indicating that the mixed bimerons-bubbles are more isotropic, as compared with disconnected labyrinths. With increasing the electric field, the structure factor then exhibits a ring which is isotropic (see E=48×10^7V/m and P=32 in Fig. S5 for example), characterizing the bubble phase. For a larger electric field (see E=84×10^7V/m and P=32 in Fig. S5), the ring of the structure factor changes to some discrete points, reflecting that the size of bubbles decreases. Further increasing the electric field (see E=96×10^7V/m and P=32 in Fig. S5), the structure factor exhibits a dark blue ring which is isotropic (*i.e.,* dipolar moments distribute uniformly in the real space), indicating that the phase evolves to the dipolar liquid phase. As similar to CMC, for a very large electric field (E=150×10^7V/m, P=32), there is only one central red peak for the structure factor, as consistent with the formation of a monodomain (along the $+z$ direction) in the real space.

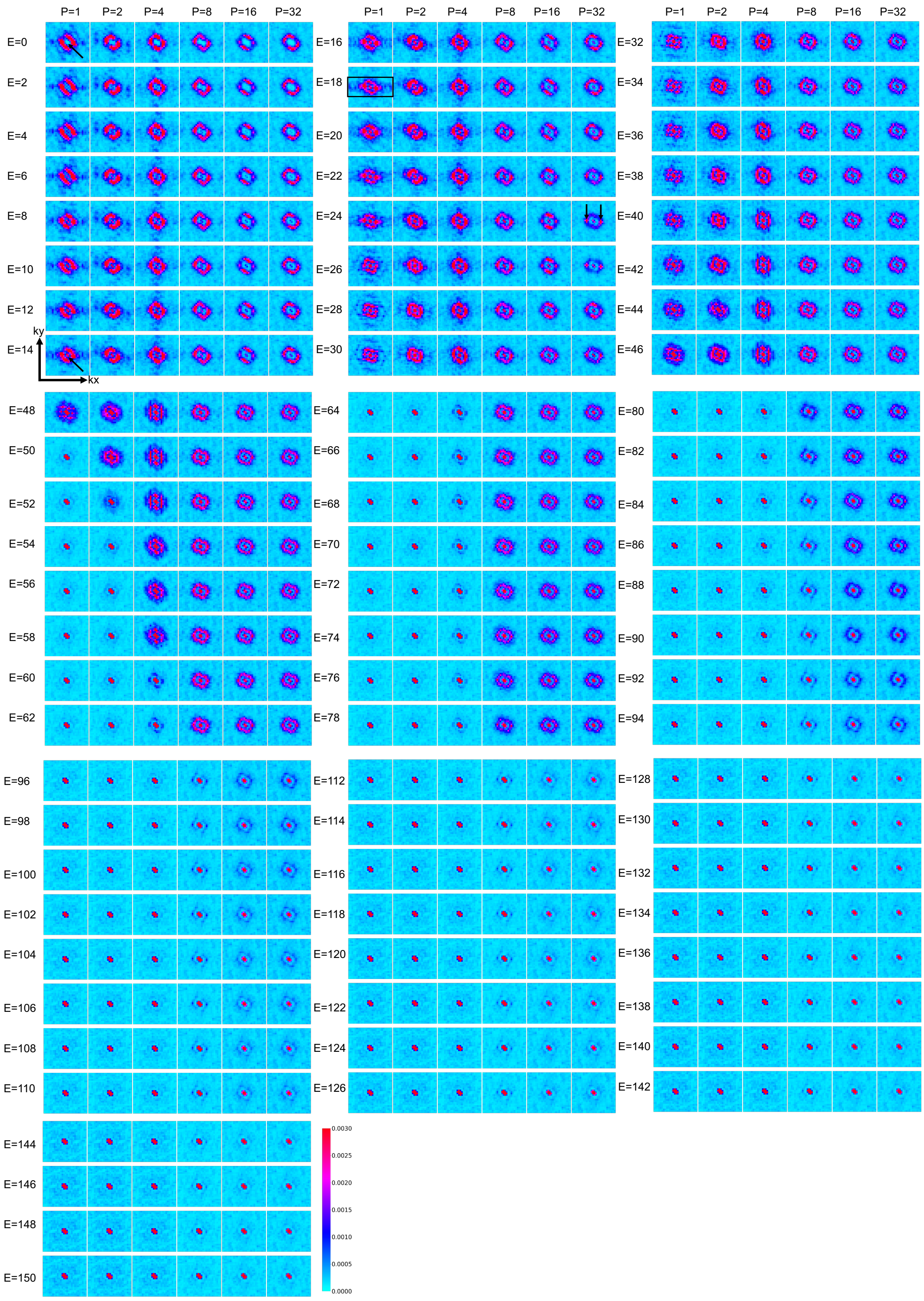

FIG. S5: The structure factor of topological patterns for all electric fields (range from 0×10^{7}V/m to 150×10^{7}V/m, with steps of 2×10^{7}V/m) and Trotter number (P=1, 2, 4, 8, 16, 32), as calculated in a 26×26×5 supercell.

Supplementary Note 6. The zeroth Betti number for different electric fields and Trotter numbers

Fig. S6 shows the calculated zeroth Betti number β_0 (which characterizes the density of the domains) [3] of topological patterns found for different electric fields and Trotter numbers. Here, we give a brief introduction for the Betti number. In algebraic topology, the Betti numbers are used to distinguish topological spaces based on the connectivity of n-dimensional simplicial complexes[4]. The kth Betti number refers to the number of k-dimensional holes on a topological surface. A "k-dimensional hole" is a k-dimensional cycle that is not a boundary of a $(k+1)$-dimensional object. The first few Betti numbers have the following definitions for 0-dimensional, 1-dimensional, and 2-dimensional simplicial complexes: β_0 is the number of connected components (which we used in our work); β_1 is the number of one-dimensional or "circular" holes; β_2 is the number of two-dimensional "voids" or "cavities". Since β_0 grows with the supercell size, we renormalize it with respect to the area of the supercell. Specifically, for our case, the Betti number is divided by 26×26=676 since we use a 26×26×5 supercell. For P=1 (see black line points), with electric field less than 14×10^7V/m, the states are labyrinths (Phases I and II in Fig. 2a of main text) and possess small β_0. Further increasing electric field (from 14×10^7V/m to 24×10^7V/m) in the classical simulations results in a β_0 that progressively increases, since more labyrinths are broken to form the Phase III that consists of mixed bimerons-bubbles. From 24×10^7V/m to 48×10^7V/m with P=1, β_0 reaches a maximum value that is characteristic of the bubble phase. When the electric field excesses 48×10^7V/m in the CMC simulations, the bubble phase starts to disappear in favor of a monodomain, which exhibits zero value for β_0. Furthermore, turning on the zero-point phonon vibrations by increasing P from 2 to 32, one can clearly see that there is (i) a right shift of the boundary between the bubble phase and monodomains; (ii) the electric-field region of the bubble phase broadens; (iii) QFs, for P larger or equal than 16, result in the formation of the bubble liquids and dipolar liquid phase which allow the system to continuously evolve from the bubbles to monodomains states; and (iv) When the

electric field is larger than 142×10^{7}V/m with P=32, monodomains are formed and β_0 is annihilated.

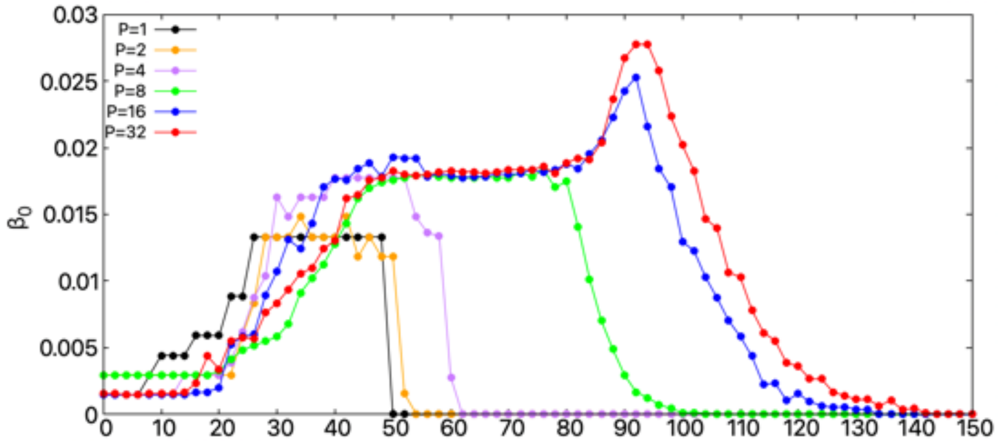

FIG. S6: The zeroth Betti number β_0 as a function of electric field for Trotter number P=1, 2, 4, 8, 16, 32, as calculated using a 26×26×5 supercell.

Supplementary Note 7. Schematic diagram of quantum critical fluctuations

At finite temperature, both thermal fluctuations and quantum fluctuations exist. The amplitude of thermal fluctuations increases with increasing temperature. As a result, thermal fluctuations dominate at high temperatures. In contrast, at very low temperatures, the key role is passed on to quantum fluctuations. The amplitude of the latter can also be controlled by an additional external parameter that is different from the temperature. In our case, such parameter is the external electric field.

The amplitude of quantum fluctuations becomes particularly high in the vicinity of the so-called quantum critical point. Such point corresponds to the critical value of the control parameter at 0K for which quantum fluctuations trigger a spontaneous symmetry breaking. At finite, and yet, low temperatures, the critical behavior is expected not at a single value but rather over a range of values of the external control parameter. Such critical region widens upon increasing temperature as shown in Fig. S7a.

To verify the quantum critical behavior, we conducted calculations at 40K, 35K, 30K, 25K and 15K (Note we cannot do lower temperature case since it will require even larger trotter number, and thus time-consuming calculations, to converge the results). In fact, the results for these temperatures of 40K, 35K, 30K, 25K and 15K already give the same qualitative results: a quantum critical region does locate in-between phase IV' (bubble liquids) and phase IV'' (dipolar liquids). Let us also emphasize that, for a finite temperature, the quantum critical point at 0K evolves to be a quantum critical region that grows in size as the temperature increases (see Fig. S7a). In our case, the quantum critical region can be identified as a region involving Phase IV' and IV'' (that range in-between phase IV, for which bubbles whose zeroth Betti number almost do not change as the electric field increases, and the monodomain of Phase V). More precisely, we plot in Fig. S7b the zeroth Betti number as a function of electric field and the black and red double-headed arrows provides a measure of the quantum critical region, for T=15K and T=40K cases, respectively. One can indeed see that T=40K has a wider quantum critical region than T=15K. From Fig. S7b, one can further see that the quantum critical region continuously shrinks as the temperature decreases from 40K to 15K, as consistent with Fig. S7a. Thus, we can expect that the quantum critical region will become a quantum critical point as the temperature reaches to 0K. However, simulating the 0K properties, is not feasible.

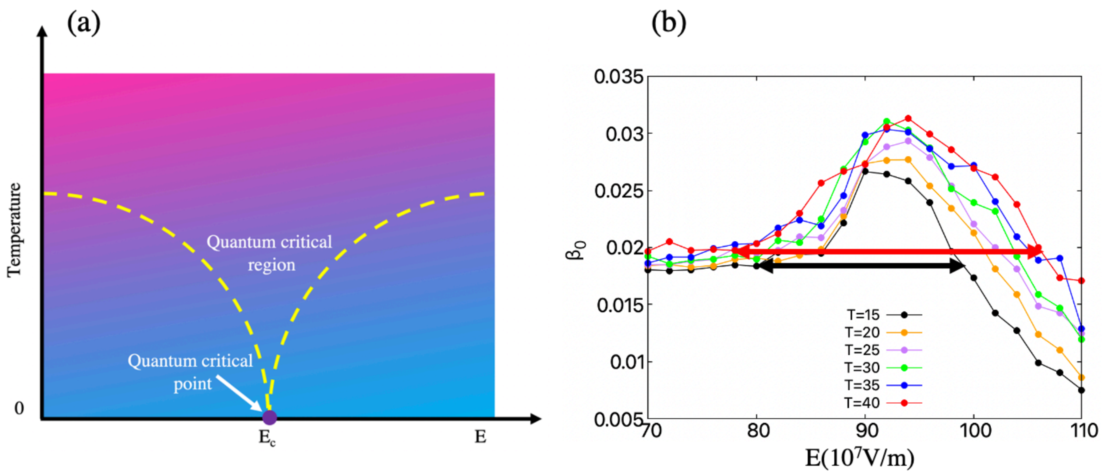

FIG. S7: (a) Schematic diagram of a quantum phase transition. In our case, the x axis

represents the direct current electric field along the z-direction. (b) The zeroth Betti number as a function of electric fields. Different colors represent different temperature cases. Here, the trotter number is P=32. The black and red double-headed arrows provide a measure of the quantum critical region for T=15K and T=40K cases.

Supplementary Note 8. Dielectric response from CMC and PI-QMC

We also calculated the dielectric properties for CMC and PI-QMC cases. Fig. S8a shows the total polarization (P) along the out-of-plane direction as a function of electric field for CMC. The dielectric coefficient χ_{33} is then obtained as the derivative of P with respect to that field. From E=0×10^{7}V/m to E=48×10^{7}V/m, P increases with increasing electric field. Near E=48×10^{7}V/m, the jump of P indicates the phase transition between bubbles and monodomains, and is accompanied by a change in the dielectric coefficient χ_{33} (Fig. S8c). For the monodomains (48 × 10^{7}V/m to 150 × 10^{7}V/m), P increases with increasing electric field but its slope slightly decreases with increasing electric field – which thus explains why χ_{33} gradually and concomitantly decreases (in a quadratic behavior).

For the PI-QMC case (with P=32), the total polarization (P) and dielectric coefficient (χ_{33}) as a function electric field are shown in Fig. S8b and 8d, respectively. Two striking changes of the dielectric coefficient χ_{33}occur. The first one characterizes the transition between disconnected labyrinths and dynamic stripes near E=22×10^{7}V/m. The second one occurs within the quantum critical region involving the bubble liquids and dipolar liquids. For fields above this quantum critical region, the χ_{33} of the dipolar liquid phase decreases (with a linear behavior) as the electric field increases. Comparing the dielectric coefficient χ_{33} in Fig. S7c (CMC) and Fig. S8d (PI-QMC), one can see that QFs have the tendency to decrease the dielectric susceptibility for common phases.

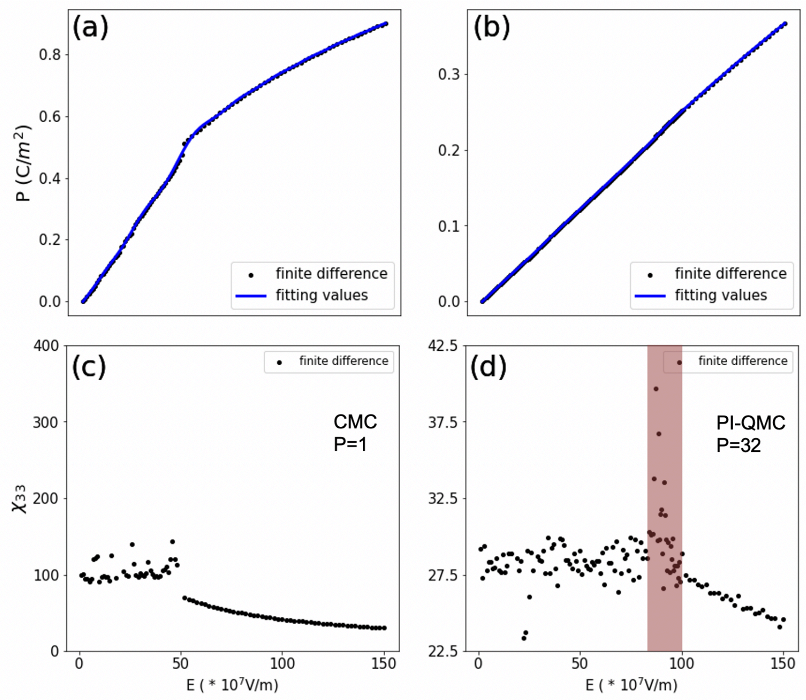

FIG. S8: The polarization (P) as a function of electric field for CMC (P=1) (a) and PI-QMC (P=32) (b) simulations. The dielectric coefficient (χ_{33}) as a function of electric field for CMC (c) and PI-QMC (d). The dielectric coefficient (χ_{33}) is calculated by the field-derivate of the total polarization P. The red region in (d) represents the quantum critical fluctuations region.

[1] I. Kornev, H. Fu, and L. Bellaiche, Physical review letters **93**, 196104 (2004).

[2] B. Berg and M. Lüscher, Nuclear Physics B **190**, 412 (1981).

[3] V. Sofonea and K. Mecke, The European Physical Journal B-Condensed Matter and Complex Systems **8**, 99 (1999).

[4] Barile, Margherita and Weisstein, Eric W. "Betti Number." From MathWorld--A Wolfram Web Resource. https://mathworld.wolfram.com/BettiNumber.html
