# Supplementary material for: Quantum criticality at cryogenic melting of polar bubble lattices
Source: Nat Commun. 2023 Nov 30;14:7874. doi: 10.1038/s41467-023-43598-0 (PMC10689468; doi:10.1038/s41467-023-43598-0)
Supplement: Supplementary file 3 — Description of Additional Supplementary Files [file 41467_2023_43598_MOESM3_ESM.docx]

Supplementary Video 1


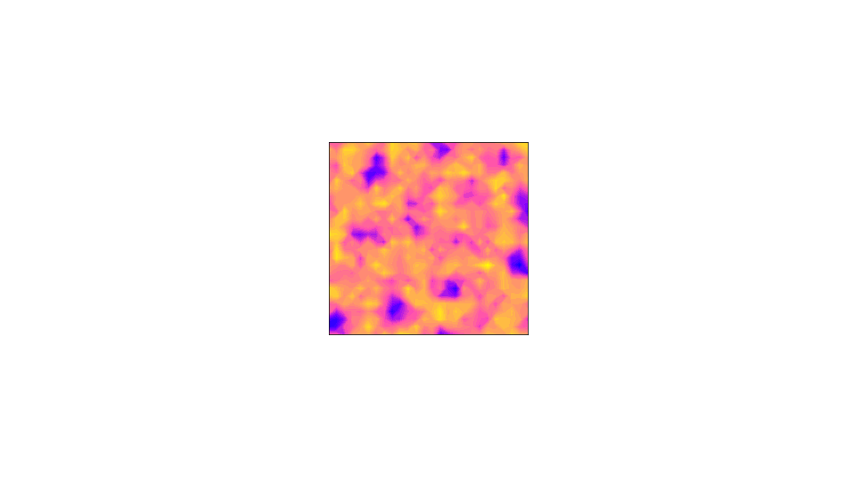


Description: The evolution of the bubble liquids (E=90$\times$10^7^V/m) as a function of

MC steps (at 20 K for P=32)

Supplementary Video 2


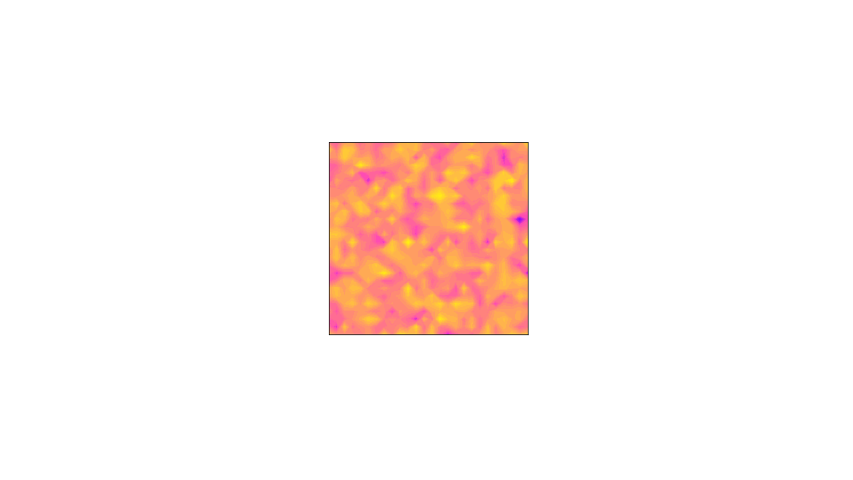


Description: The evolution of the dipolar liquid phase (E=114$\times$10^7^V/m) as a function of MC steps (at 20 K for P=32).

Supplementary Video 3


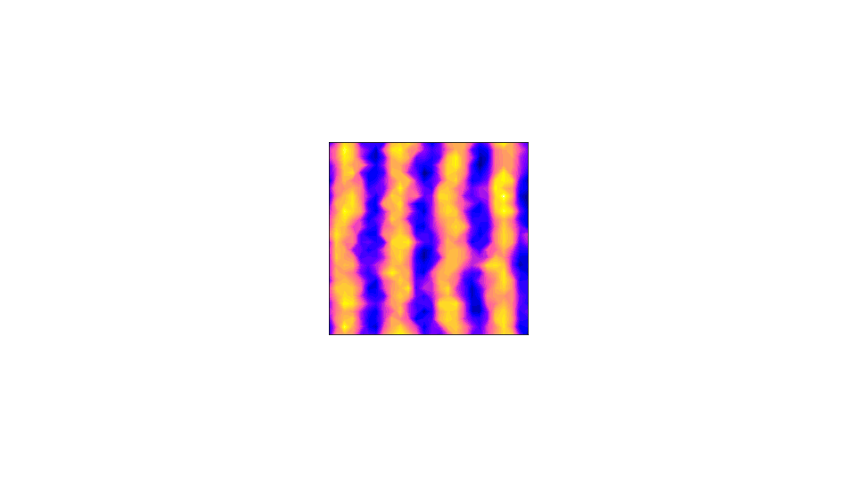


Description: The evolution of the dynamic stripes (E=26$\times$10^7^V/m) as a function of MC steps (at 20 K for P=32).
